# Central retinal artery occlusion as a result of symptomatic patent foramen ovale

**DOI:** 10.22336/rjo.2023.12

**Published:** 2023

**Authors:** Haroon Tayyab, Faiqa Binte Aamir, Salman Kirmani

**Affiliations:** * Department of Ophthalmology and Visual Sciences, The Aga Khan University Hospital, Karachi, Pakistan; ** Division of Women and Child Health, The Aga Khan University Hospital, Karachi, Pakistan

**Keywords:** central retinal artery occlusion, patent foramen ovale, transesophageal echocardiography

## Abstract

**Objective:** The objective of this case report is to highlight the importance of patent foramen ovale (PFO) as a potential cause of central retinal artery occlusion (CRAO).

**Methods:** A teenage girl presented with a sudden painless onset of vision loss in the right eye, which was accompanied by frontal headaches and vertigo. The patient was referred to the Ophthalmology Department, where subsequent examination revealed a best corrected visual acuity of 20/ 400 and a positive relative afferent pupillary defect (RAPD) in the right eye. Fundoscopy and optical coherence tomography confirmed the diagnosis of central retinal artery occlusion following which investigations to rule out hematologic, vascular, and cardiac causes were performed.

**Results:** Transoesophageal echocardiography revealed PFO as the cause of this cryptogenic stroke. All the necessary blood testing work was performed (complete blood counts, erythrocyte sedimentation rate, C-reactive protein, lipid profile, homocysteine levels, prothrombin time, activated partial thromboplastin time, international normalized ratio, liver, renal and thyroid function tests, antinuclear antibodies, anti-smooth muscle antibodies, anti-mitochondrial antibodies, p-ANCA, c-ANCA, anti-cardiolipin antibodies, protein C, Protein S, activated protein C resistance, anti-thrombin III, VDRL, antibodies for viral retinitis, angiotensin converting enzyme, Mantoux test, detailed urine and electrolyte reports). Transoesophageal echocardiography revealed right to left shunt.

**Conclusions:** This case along with other reported evidence in literature support the strong connection between PFO and CRAO. Closure of symptomatic PFO may result in prevention of severe visual loss.

**Abbreviations:** CRAO = central retinal artery occlusion, PFO = patent foramen ovale, RAPD = relative afferent pupillary defect, BCVA = best corrected visual acuity, OCT = Optical coherence tomography, IOP = Intraocular pressures, TTE = transthoracic echocardiography, HM = hand motion, TEE = transesophageal echocardiogram

## Introduction

Sudden unilateral loss of vision is an ophthalmic emergency and central retinal artery occlusion (CRAO) is one of the leading important causes. It is analogous to acute stroke of the eye and has an incidence of 1 in 100,000 people [**1**]. As CRAO affects the inner retinal layers (ganglion cell layer, nerve fiber layer), it has devastating functional consequences in the form of severe visual impairment. Unfortunately, current medical science has no definite treatment protocol to reverse the ischemic effects of CRAO. This is unlike stroke, in which treatment protocols are well established [**2**]. Etiologies for CRAO vary with the age groups and persisting risk factors but often, it is associated with rare conditions as well. One such rare emerging association is patent foramen ovale (PFO). It is a benign condition (25% of population), in which embryologic interatrial communication persists even after the neonatal year, which can lead to right to left sided shunt. In utero, PFO allows right to left shunt, but soon after birth and with establishment of pulmonary circulation, the PFO closes due to a drop in pulmonary pressure and an increase in left atrial pressure as compared to the right. Thus, a persistent PFO, can rarely be responsible for embolic episodes resulting in stroke and CRAO [**3**-**5**]. Other published cases have also reported other ocular problems including branch retinal artery occlusion and optic neuropathy to be associated with PFO, which has also been reported as a causative factor, leading to migraine and decompression sickness in deep sea divers [**6**].

This case report describes a case of an 11-year-old girl with sudden painless loss of vision in the right eye, which was accompanied with sudden onset frontal headaches and vertigo. Investigations to rule out hematologic, vascular, and cardiac causes were performed and PFO, diagnosed on echocardiography, was identified as the cause of CRAO.

## Case presentation

**Fig. 1:**
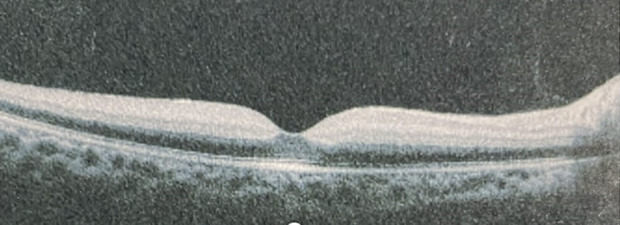
OCT scan of the right eye shows hyperreflectivity of inner retinal layers

**Fig. 2:**
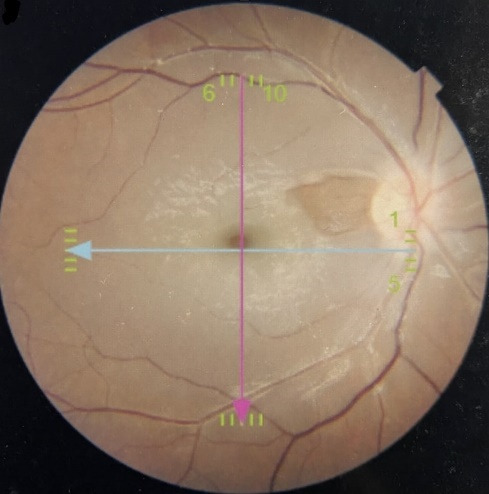
Colored fundus picture of the right eye showing cherry red spot

A teenage girl presented at a tertiary care hospital in Pakistan with complaints of sudden profound unilateral painless loss of vision in the right eye for five days. It was preceded by a one-month history of frontal headaches, which were sudden in onset and mild to moderate in intensity, being accompanied by vertigo attacks. Apart from moderate myopia, there was no significant ocular history. She was not taking any medications and the past medical history was also insignificant. At the time of presentation, her best corrected visual acuity (BCVA) was 20/ 400 in the right eye and 20/ 30 in the left eye. A relative afferent pupillary defect (RAPD) was present in the right eye, but no significant findings were noted in the anterior chamber and vitreous in both eyes. Intraocular pressures (IOP) were 14 mmHg and 16 mmHg in the right and left the eye respectively. On detailed retinal exam, a big cotton wool spot was noticed in the right eye, resembling CRAO, based on which the diagnosis of CRAO in right eye was made. The left retinal exam was normal. Optical coherence tomography (OCT) was performed, which also confirmed the diagnosis of right CRAO with hyperreflectivity of inner retinal layers (**Fig. 1**). Fundus picture showed cherry red spot with edema of inner retinal layers (**Fig. 2**).

Because of this very unusual presentation, all the necessary blood testing work was performed (complete blood counts, erythrocyte sedimentation rate, C-reactive protein, lipid profile, homocysteine levels, prothrombin time, activated partial thromboplastin time, international normalized ratio, liver, renal and thyroid function tests, antinuclear antibodies, anti-smooth muscle antibodies, anti-mitochondrial antibodies, p-ANCA, c-ANCA, anti-cardiolipin antibodies, protein C, Protein S, activated protein C resistance, anti-thrombin III, VDRL, antibodies for viral retinitis, angiotensin converting enzyme, Mantoux test, detailed urine and electrolyte reports) and apart from a low hemoglobin level of 10 mg/ dl, all reports came out normal.

We also performed a necessary radiological exam including magnetic resonance imaging of brain and carotid arterial doppler and the findings were insignificant. Visual evoked potential test was also performed, which showed right optic nerve dysfunction that was nonreactive to N-75, P-100 and N-145 waveforms.

Afterwards, a pediatric cardiology consult was sought to rule out any embolic etiology of cardiac origin. Her general cardiac exam was normal, with blood pressure of 130/ 85 mmHg and pulse of 88 beats/ minute. A transthoracic echocardiography (TTE) was performed, which showed PFO with ejection fraction of 69%. This was later confirmed with a positive provocation bubble study during TTE, showing a right to left shunt. This was highly suggestive of a large PFO. No other potential causes for the retinal artery embolus were identified at any point. 

Given the potential of future ophthalmic and cerebral emboli risk, a percutaneous closure of PFO was advised along with long term anti-coagulation therapy. A detailed informed consent was obtained from parents and the girl was informed regarding the report of this rare clinical scenario. 

After one year of follow-up, the patient did not report of any further embolic events, and she maintained hand motion (HM) visual acuity in the right eye. 

## Discussion

PFO has been reported in a few case reports, in which the patients presented with sudden acute loss of vision attributed to CRAO or BRAO. These reports did not find any additional risk factors for CRAO and have mostly reported such cases in young adults [**7**]. PFO has been found in 20-25% of adults and recent evidence has suggested a positive correlation between right to left shunt mediated by PFO and risk of stroke and migraine [**8**]. Wieder et al. reported a similar case of PFO with right to left shunt and CRAO. They also conducted a review of literature and found seven such other cases, in which PFO has been implicated as a causative factor of CRAO. In their review, they cited transesophageal echocardiogram (TEE) as being more sensitive in diagnosing PFO as compared to TTE [**9**,**10**]. Our case also reported a similar occurrence of CRAO and PFO.

Similarly, Zhu et al. described a case of a 46-year-old male with history of amaurosis fugax in one eye for 20 minutes followed by acute painless and profound loss of vision. The patient did not have any other risk factors of CRAO apart from PFO documented on TEE and blood split-flow from right to left atrium when performing Valsalva breath during the Foaming test [**11**].

Retinal artery occlusion is a visually devastating condition, in which acute obstruction of central retinal artery leads to irreversible ischemia of the retinal ganglion cells [**12**]. Historically, the time interval between complete CRAO and irreversible retinal ischemia has been found to be 90-240 minutes and all efforts shall be done to dissolve or dislodge the thrombus. However, recent literature quotes the window period between occlusion to infarction to be 12-15 minutes. This is the reason there is so strong evidence of efficacy of any intervention in case of CRAO [**13**].

PFO can be compounded with other pathologies when associated with CRAO. This includes a case, in which atrial septal aneurysm associated with PFO resulted in CRAO in a young adult [**14**]. Similarly, a case by Zhu et al., in which PFO acted as a source of paradoxical emboli in presence of internal carotid hypoplasia and resulted in CRAO, was recently reported [**11**]. Similarly, PFO has also been associated with branch retinal artery occlusion, which is a relatively visually forgiving event, but the pathogenesis is the same [**4**,**5**]. The causal relationship between PFO and thromboembolism has not been fully established and all proposed mechanisms are complex. These include emboli forming in atrial septum, transient arrythmias leading to thrombus formation and paradoxical emboli from peripheral venous system. This is the reason a thorough cardiologic examination shall be performed in case a calcific embolus is detected in retinal circulation and TEE is more sensitive in detecting PFO as compared to TTE [**14**].

## Conclusion

With this interesting case report, we conclude that PFO shall be considered in cases of CRAO and branch retinal vein occlusion (BRAO) in young patients, in which no other risk factors can be identified. It is imperative to refer these patients to the cardiology team so that necessary intervention can take place to prevent future thromboembolic events. This case report also highlights the merit of elective treatment of PFO in asymptomatic patients.

**Conflict of Interest Statement**

The authors state no conflict of interest.

**Informed Consent and Human and Animal Rights statement**

Informed consent has been obtained from the parents of the patient included in the study.

**Authorization for the use of human subjects**

Ethical approval: The procedures followed were in accordance with the ethical standards of the institutional committee on human experimentation of The Aga Khan University Hospital, Karachi, Pakistan and with the Helsinki Declaration of 1975, as revised in 2000 and 2008.

**Acknowledgements**

None. 

**Sources of Funding**

None. 

**Disclosures**

None.

**Contribution**

All authors contributed equally to this article. 
